# Mid- and long-term associations between food insecurity and sarcopenia

**DOI:** 10.1007/s40520-025-02999-5

**Published:** 2025-03-13

**Authors:** Aarón Salinas-Rodríguez, Vanessa De la Cruz-Góngora, Betty Manrique-Espinoza

**Affiliations:** National Institute of Public Health, Cuernavaca, Morelos Mexico

**Keywords:** Sarcopenia, Food insecurity, Older adults, SAGE-Mexico

## Abstract

**Background:**

Sarcopenia is a complex geriatric syndrome characterized by progressive and generalized loss of skeletal muscle mass, muscle strength, and physical performance. Nutritional factors, including food insecurity, have been reported to be important in the development of sarcopenia. However, evidence on the relationship between sarcopenia and food insecurity is limited, especially with longitudinal data.

**Aims:**

This study aimed to examine the longitudinal association between sarcopenia, severe sarcopenia, and food insecurity in a nationally representative sample of older adults in Mexico.

**Methods:**

We used data from the four waves (2009, 2014, 2017, 2021) of the World Health Organization Study on Global Ageing and Adult Health in Mexico. The sample consisted of 1,484 older adults aged 50 years or older. Sarcopenia was defined according to the criteria of the European Working Group on Sarcopenia in Older People. Food insecurity was assessed with two questions related to frequency of eating less and hunger due to lack of food in the last 12 months.

**Results:**

Moderate (OR = 1.13; 95%CI: 1.09–1.20) and severe food insecurity (OR = 1.19; 95%CI: 1.11–1.27) significantly increased the longitudinal rates of sarcopenia or severe sarcopenia. Meanwhile, the incidence of severe food insecurity increased the cumulative incidence rate of sarcopenia and severe sarcopenia (OR = 1.91; 95%CI: 1.24–2.94).

**Discussion:**

Since food insecurity is a modifiable structural factor, the implementation of specific programs to alleviate its deleterious consequences is warranted.

**Conclusions:**

This study shows that moderate and severe food insecurity are associated with an increase in the rates of sarcopenia and severe sarcopenia over time.

**Supplementary Information:**

The online version contains supplementary material available at 10.1007/s40520-025-02999-5.

## Introduction

The aging process is often accompanied by changes in body composition that decrease lean mass and skeletal muscle functionality due to increased adiposity and redistribution of fat from subcutaneous regions to the intra-abdominal area [1]. Sarcopenia is a condition associated with these changes characterized by a progressive and generalized alteration of skeletal muscle mass [2]. It is also considered a geriatric syndrome of multifactorial etiology whose prevalence increases with age [3].

Sarcopenia is a major challenge for public health policies due to its high prevalence and negative consequences on mobility, morbidity, functional dependency, mortality [2] and the quality of life of older adults [4]. In low- and middle-income countries (LMICs), a prevalence of 13.5% has been reported [5], while in Mexico, the prevalence ranges between 9% and 33% [6].

Multiple factors have been reported as potential causes of sarcopenia, which include biological traits (oxidative stress, inflammation), disease-related conditions, behavioral or lifestyle aspects, and sociodemographic characteristics [7]. Notably, nutritional factors have been identified as modifiable elements associated with sarcopenia [8, 9], including an adequate quality diet, sufficient intake of protein, vitamin D, and antioxidant nutrients [10]. In turn, food insecurity is one of the main factors that most affect access to a good quality diet [11].

Food insecurity (FI) refers to limited or uncertain access to sufficient food to achieve an active and healthy life. It is a public health problem and a main social determinant of health that affects populations of all ages [12]. However, despite being a persistent, growing, and clinically relevant problem for the older adult population, research on the consequences of FI on vital health outcomes for this population group has been scarce [13]. In fact, FI is a problem that particularly affects older adults in low- and middle-income countries [14]. One study conducted in six LMICs reported that the prevalence of moderate or severe FI was 11.7% [5], while for Mexico a prevalence of 20.8% has been reported [15].

Although evidence on the relationship between FI and sarcopenia is limited, recent studies suggest that older adults who experience FI are more likely to have lower muscle strength [16], lower physical performance [17], and higher likelihood of sarcopenia [5]. Although these results have been mainly generated with cross-sectional data.

Given the magnitude and challenges that FI represents for low- and middle-income countries, as well as the high prevalence of sarcopenia and its relationship with the quality of life and well-being of older adults, this study aimed to examine the longitudinal relationship between both conditions. Two hypotheses will be tested using data from a cohort study of older adults in Mexico. First, rates of sarcopenia and severe sarcopenia increase differently according to the severity level of FI during a 12-year follow-up period. Second, severity levels of FI are associated with higher incidence rates of sarcopenia and severe sarcopenia during a 4-year follow-up.

## Methods

### Population and sample

Data from the four waves (2009, 2014, 2017, 2021) of the World Health Organization (WHO) Study of Global Ageing and Adult Health (SAGE) in Mexico were used. SAGE is a multinational longitudinal study based on nationally representative samples of people aged 50 years and older in six countries: China, Ghana, India, Mexico, Russia, and South Africa. Details of its design have been previously published [18]. The design and sample of the SAGE-Mexico study have also been previously described [19]. Briefly, SAGE-Mexico is an open cohort with national representativeness, including a follow-up and refreshment samples at each wave. Wave 1 (baseline data) was collected in 2009 with 2,404 respondents. Wave 2 was conducted in 2014 with 2,033 interviews (plus 618 new individuals). Wave 3 was carried out in 2017 with 1,791 participants (plus 255 new interviews), and Wave 4 in 2021 with 1,607 older adults (plus 354 new individuals). The analytical sample consisted of 1,484 older adults with all wave measurements (Fig. S1 in supplementary material). Baseline differences were observed between the final sample and excluded participants. Older adults without follow-up measurements were older, with a higher prevalence of sarcopenia and smoking, mostly women and mainly from rural areas (*p* < 0.05).

### Outcome variable

Sarcopenia. It was defined according to the criteria specified by the European Working Group on Sarcopenia in Older People 2 [20] and previous studies using SAGE-Mexico data [6]. Specifically, sarcopenia was determined according to the following criteria:

- Weak grip strength. It was determined as < 27 kg for men and < 16 kg for women using the average value of the two grip measurements of the dominant hand.
- Low skeletal muscle mass. Skeletal muscle mass (SMM) was calculated as the appendicular skeletal muscle mass (ASM) according to the equation proposed by Lee et al. [21]. Additionally, skeletal muscle mass index (SMI) was obtained by dividing ASM by body mass index (BMI) [22]. Then, low SMM (defined as the presence of low SMI) was established by the lowest quintile of SMI based on sex-stratified values.
- Slow walking speed. It was defined as the lowest quintile of walking speed (meters/second) based on height, age, and sex-stratified values.

Finally, sarcopenia was defined if weak grip strength and low skeletal muscle mass were present, and severe sarcopenia if the three criteria were met.

### Main exposure

Food insecurity. The following two questions, related to frequency of eating less and hunger due to lack of food, were used to operationalize this variable: “In the last 12 months, how often did you ever eat less than you felt you should because there wasn’t enough food?” and “In the last 12 months, were you ever hungry, but didn’t eat because you couldn’t afford enough food?”. Both questions had response options: every month (code = 1); almost every month (code = 2); some months, but not every month (code = 3); only 1 or 2 months (code = 4); never (code = 5). These questions were adapted from similar items in food security questionnaires, such as the US Household Food Security Survey Module, the National Health and Nutrition Examination Survey (NHANES) Food Security Module and the Mexican Health and Nutrition National Survey. Based on previous studies using SAGE data [23–26], those who answered 1 to 3 to both questions or 1 to either question were classified as severely food insecure. Those who did not meet the criteria for severe food insecurity but answered 2 to 4 to either question were classified as moderately food insecure. Furthermore, those who answered 5 to both questions were classified as food secure.

### Covariates

Socioeconomic. Sex (female = 1), age, number of years of formal education, marital status (married/cohabiting versus single/widowed/divorced), employment status (paid job = 1), and area of residence (rural = 1). Household socioeconomic status (SES) was obtained using the standard WHO approach to estimate permanent income from durable goods ownership, housing characteristics (type of floor, wall, and kitchen), and access to services such as water, sanitation, and electricity [27]. SES was included as a continuous variable, with higher values indicating higher SES.

Health. Multimorbidity was included as a dichotomous variable defined as the presence of two or more chronic non-communicable diseases from the 12 chronic conditions included in the SAGE-Mexico study. These diseases’ lists and operational definitions have been previously published [28]. Body mass index (BMI) was calculated using weight (kg) and height (cm) (BMI = Weight (kg)/Height (m^2^)) and was incorporated into the analysis as a continuous variable. C-reactive protein values (mg/L) were included as a potential marker of inflammation.

Health-related lifestyles. Physical activity was assessed with the Global Physical Activity Questionnaire (GPAQ), which classifies older adults into three categories (low, moderate, and high physical activity) based on reported time spent in moderate or vigorous activities during work, recreation/leisure time, and transportation [29]. Regarding tobacco use and alcohol consumption, older adults were asked if they had ever used tobacco or alcohol and if participants answered affirmatively, the frequency of use was recorded. With this information, tobacco use was categorized as never; ever smoked, no longer; current smoker, not daily; current smoker, daily; and alcohol consumption as never; ever drinker, no longer; current drinker, low risk; current drinker, high risk. Fruit and vegetable consumption (servings per day) and sedentary behavior (daily hours of sitting) were obtained by self-report.

### Statistical analysis

Baseline characteristics are presented as percentages and means (standard error) as appropriate. Health and sociodemographic characteristics related to FI categories were compared using Chi-square or ANOVA tests.

### Longitudinal association of FI levels and sarcopenia

Given that the response variable has three ordinal categories (no sarcopenia, sarcopenia, and severe sarcopenia) and the repeated measurements for each individual, a mixed-effects ordinal logistic regression model was fitted. Since we aimed to test the hypothesis that the rates of sarcopenia and severe sarcopenia increase differently according to the level of severity of FI during a 12-year follow-up period, the model included a multiplicative interaction term between the FI variable (for each of their categories) and the total follow-up time observed for each individual. For this first analytical approach, data from the four waves of SAGE-Mexico were used. In addition, the model specification included the wave-specific centered age to minimize collinearity problems and separate the effects of time and age.

### Association between severity of FI with incidence rates of sarcopenia and severe sarcopenia

The two most recent waves of SAGE-Mexico (2017 and 2021) were used to test the hypothesis that levels of FI severity are associated with higher incidence rates of sarcopenia and severe sarcopenia during a mean 4-year follow-up. Since the interest was in modeling (cumulative) incidence, the model was specified as follows in this analytical approach. First, older adults with sarcopenia at baseline (wave 3, 2017) were excluded, so only those “free” of the disease were included. At the end of follow-up (wave 4, 2021), the incidence rate of sarcopenia (moderate or severe) was compared according to the degree of baseline FI, controlling for the baseline values of the covariates.

### Sensitivity analyses

An additional model was fitted to determine whether the incidence of IF (moderate or severe) observed between SAGE-Mexico waves 3 and 4 was associated with the incidence of sarcopenia or severe sarcopenia. For this analysis, IF was operationalized into the following categories: 0 = No IF, 1 = Incidence of moderate IF, and 2 = Incidence of severe IF. Baseline values of covariates were also adjusted for in this model.

Since the association between sarcopenia and FI could be bidirectional, an additional model was fitted in which the outcome was FI, and the exposure variable was sarcopenia. This was also a mixed-effects ordinal logistic regression model, given that the FI variable consists of three ordinal categories (none, moderate, severe).

Statistical analyses were performed using Stata 18.5 software (StataCorp. 2024. Stata Statistical Software: Release 18.5, College Station, TX: StataCorp LLC.). In all cases, the proportional odds assumption related to the ordinal logistic regression models was verified using the Brant test [30]. In addition to the association measures (OR), adjusted incidence rates for sarcopenia and severe sarcopenia were estimated and reported using the *margins* function in Stata. Differences were considered statistically significant if *p* < 0.05, and odds ratios (OR) and 95% confidence intervals (CI) were reported.

This study was conducted following the STROBE guidelines for reporting cohort studies (STROBE checklist is reported in Table S1 of the supplementary material).

## Results

### Baseline characteristics

Table 1 presents sociodemographic and health characteristics according to baseline FI status. The baseline prevalence of sarcopenia was 12.9%, and that of severe sarcopenia was 2.7%. A prevalence of 7.8% was observed for moderate FI, and for severe FI, 10.7%. No significant baseline differences were observed for the prevalence of sarcopenia or severe sarcopenia according to the severity level of FI (*p* = 0.70).

**Table 1:** Baseline, sociodemographic and health characteristics according to baseline food insecurity status

		Food insecurity^a^			*p*-value^*^
	Total *n* = 1484	No 81.5%	Moderate 7.8%	Severe 10.7%	
**Outcome**					
Sarcopenia	12.9	12.3	16.7	14.9	
Severe sarcopenia	2.7	2.6	2.9	2.8	0.70
**Covariates**					
Sex (female)	55.4	54.3	62.4	59.6	0.68
Age	68.2 (8.2)	68.4 (8.2)	67.4 (8.2)	67.2 (8.0)	0.49
Body Mass Index (kg/m^2^)	28.8 (5.6)	28.9 (5.5)	27.8 (5.6)	28.6 (6.5)	0.14
Multimorbidity (2 o more chronic conditions)	65.5	65.4	75.6	61.3	0.48
C-reactive protein (mg/L)	2.6 (4.3)	2.6 (4.4)	2.9 (4.9)	2.7 (3.3)	0.82
Physical activity					
Low	40.1	39.8	47.1	37.6	
Moderate	31.1	32.1	25.5	26.9	
High	28.8	28.1	27.5	35.5	0.19
Sedentary behavior (daily sitting hours)	3.0 (2.5)	3.1 (2.4)	2.9 (2.6)	2.8 (2.4)	0.39
Alcohol consumption					
Never	35.1	33.5	34.7	47.1	
Ever drinker, no longer	49.8	49.4	55.1	48.6	
Current drinker (low risk)	9.3	10.9	3.1	2.2	
Current drinker (high risk)	5.8	6.2	7.1	2.2	< 0.01
Smooking habit					
Never	63.4	62.3	74.5	63.8	
Ever smoked, no longer	17.1	17.7	10.8	17.0	
Current smoker, not daily	6.2	6.1	7.8	5.7	
Current smoker, daily	13.4	14.0	6.9	13.5	0.18
Fruits and vegetable consumption (number of portions per day)	2.6 (1.9)	2.7 (1.9)	2.2 (1.9)	2.3 (1.8)	< 0.01
Paid job	29.1	28.9	26.5	31.9	0.64
Marital status					
Single/Widowed/Divorced	36.7	35.9	39.2	40.4	
Married/Cohabitation	63.3	64.1	60.8	59.6	0.50
Schooling (years of formal education)	5.3 (4.6)	5.6 (4.7)	4.1 (3.9)	3.8 (3.6)	< 0.01
Socioeconomic status (assets index)	0.0 (1.2)	0.2 (1.1)	-0.6 (1.2)	-0.8 (1.0)	< 0.01
Dwelling area (rural)	19.4	17.4	26.7	28.6	0.34

* ANOVA or Chi-squared tests

^a^ Cells are means (std. dev.) or percentages

### Longitudinal association of FI levels and sarcopenia

**Fig. 1:**
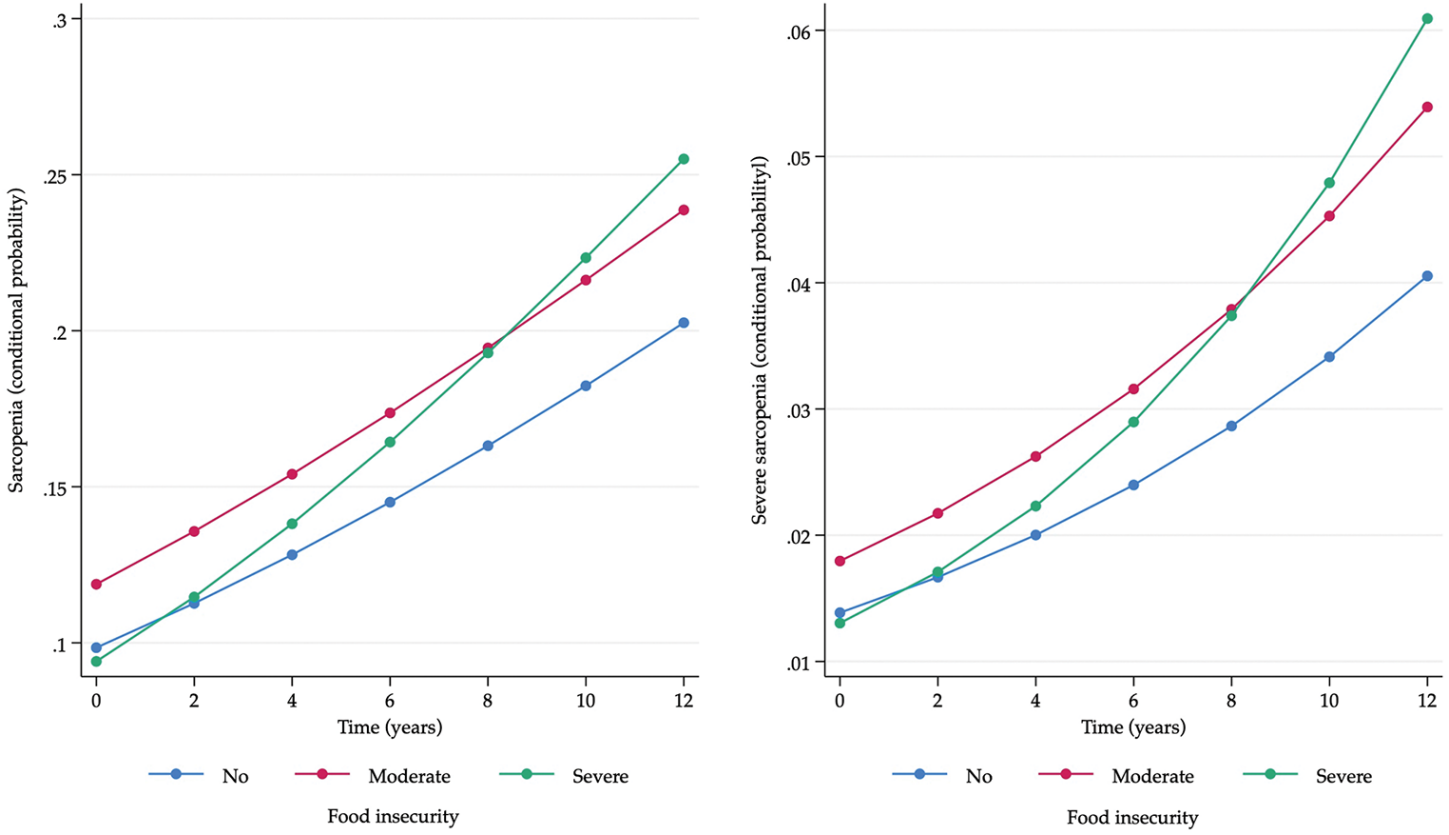
Longitudinal rates of sarcopenia and severe sarcopenia according to levels of food insecurity

The results showed that the rates of sarcopenia and severe sarcopenia increase over time and that they do so in a differentiated manner according to the severity levels of FI (overall p-value of the interaction < 0.01, Table 2, Panel A). Specifically, for older adults without FI, the increase was smaller (OR = 1.12, 95% CI: 1.10–1.15) compared to those with moderate FI (OR = 1.13, 95% CI: 1.09–1.20) and even smaller compared with those with severe FI (OR = 1.19, 95% CI: 1.11–1.27). The results of the Brant test for this model showed that the proportional odds assumption was met (*p* = 0.71) (Fig 1).

**Table 2:** Longitudinal associations of sarcopenia and food insecurity

	Sarcopenia (moderate or severe)			
**Panel A**	OR	CI 95%		p-value
**Longitudinal association**				
Interaction term time*food insecurity (*p* < 0.01)				
No food insecurity	1.12	1.10	1.15	< 0.01
Moderate food insecurity	1.13	1.09	1.20	< 0.01
Severe food insecurity	1.19	1.11	1.27	< 0.01
Brant test* (*p* = 0.71)				
**Panel B**	OR	CI 95%		p-value
**Incidence modelling**				
Baseline food insecurity (reference category: no)				
Moderate	1.38	0.76	2.52	0.30
Severe	1.80	1.08	2.98	0.02
Brant test* (*p* = 0.14)				
Incident food insecurity (reference category: no)				
Moderate	1.09	0.65	1.84	0.74
Severe	1.91	1.24	2.94	< 0.01
Brant test* (*p* = 0.19)				

* Brant test for proportional odds assumption

The graphical representation of these results is shown in Fig. 1, where the rates (expressed as conditional probabilities) of sarcopenia and severe sarcopenia are reported according to the level of FI and the follow-up time expressed annually. In both cases, a constant increase can be seen for the three FI groups (no, moderate, severe), and similar trends can be observed for the groups with no FI and with moderate FI, although with higher rates for moderate FI. On the other hand, and also in both cases, the trend for the severe FI group is the one that differs the most, with a greater increase. The overall change in the sarcopenia rates for each group (difference between the baseline and final measurement) was: with no FI 10.4%, moderate FI 12.0%, and severe FI 16.1%. For severe sarcopenia, the numbers were no FI 2.7%, moderate FI 3.6%, and severe FI 4.8%.

### Incidence rates of sarcopenia and severe sarcopenia

Table 3 shows the cumulative incidence rates (observed between waves 3 and 4 for a median follow-up period of 4 years) for the sarcopenia and FI groups. The rate for sarcopenia was 18.1% (95% CI: 16.0-20.4), and for severe sarcopenia, 1.6% (95% CI: 1.0-2.5). Meanwhile, for moderate FI, the rate was 6.8% (95% CI: 4.8–9.8), and for severe FI, 5.7% (95% CI: 3.8–8.4).

**Table 3:** Cumulative incidence rates of sarcopenia and food insecurity

Cumulative incidence^a^	Estimator	CI 95%	
Sarcopenia	18.1	16.0	20.4
Severe sarcopenia	1.6	1.0	2.5
Food insecurity			
Moderate	6.8	4.8	9.8
Severe	5.7	3.8	8.4

^a^ Cumulative incidence rate for a 4-year follow-up period (2017–2021)

The results of the ordinal logistic regression models for modeling the incidence of sarcopenia or severe sarcopenia are shown in Table 2, Panel B. In the first approach, using baseline FI values as the exposure variable, only severe FI significantly increased the incidence of sarcopenia or severe sarcopenia (OR = 1.80; 95% CI: 1.08–2.98; *p* = 0.02). Similar results were observed when using the groups defined by the incidence of FI as the exposure variable. Again, only severe FI was found to be associated with a higher incidence of sarcopenia or severe sarcopenia (OR = 1.91; 95% CI: 1.24–2.94; *p* < 0.01). For both models, the results of the Brant test showed that the proportional odds assumption was net (*p* = 0.14 in the first case and *p* = 0.19 in the second).

**Fig. 2:**
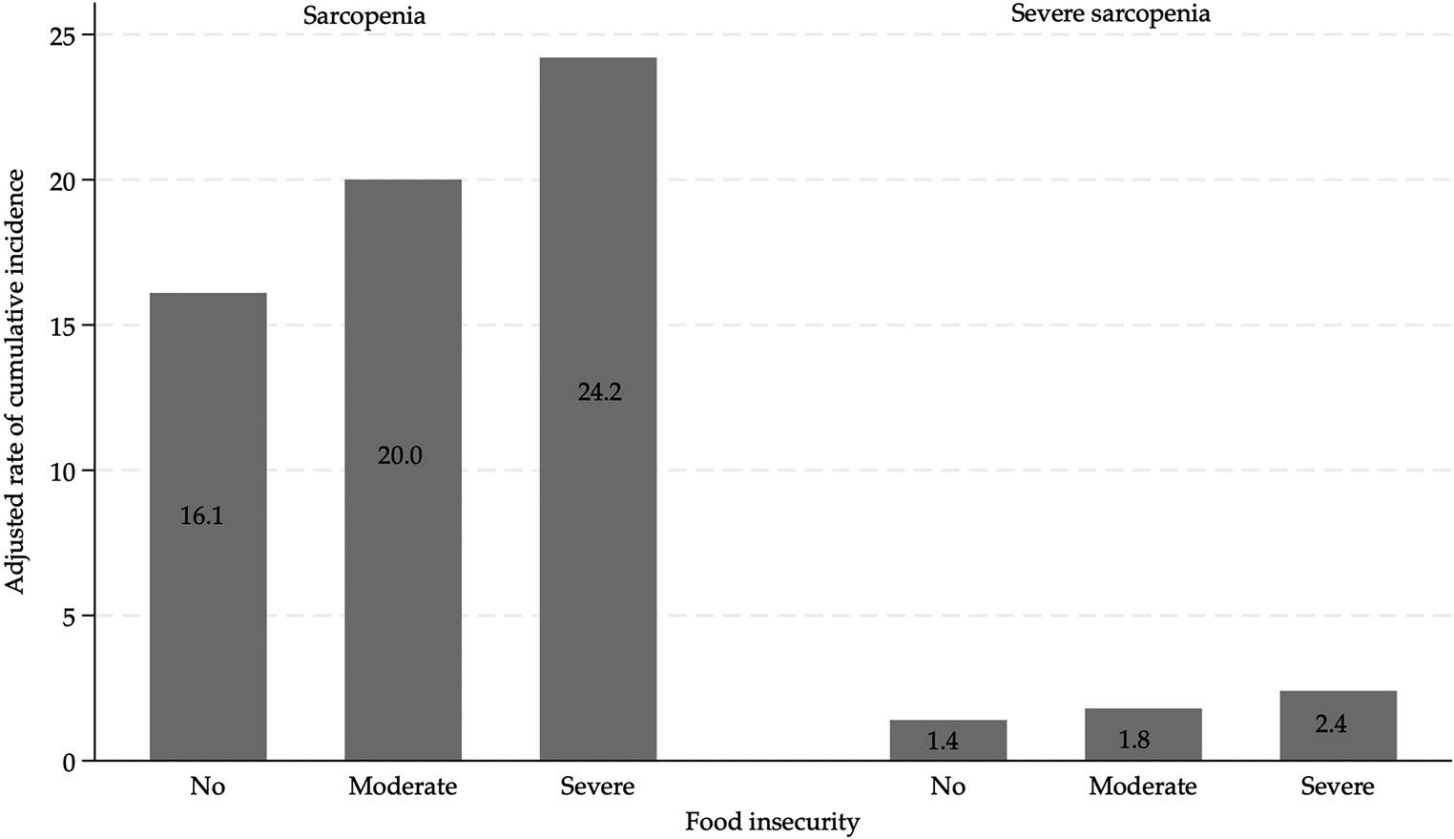
Cumulative incidence rates of sarcopenia and severe sarcopenia according to food insecurity levels

Figure 2 shows the adjusted incidence rates for sarcopenia and severe sarcopenia according to the groups defined by the incidence of FI. The results reveal that the incidence of severe FI increased the incidence of sarcopenia (24.2%) to a greater extent than moderate FI (20.0%) or the absence of FI (16.1%). The same trend was observed for severe sarcopenia, with incidence rates for severe FI at 2.4%, for moderate FI at 1.8%, and with no FI at 1.4%.

### Longitudinal reverse association of FI (outcome) and sarcopenia (exposure)

The results of the mixed-effects ordinal logistic regression model using FI as the outcome are shown in Table S2 of the supplementary material. Compared with older adults without sarcopenia, those with sarcopenia were more likely to have moderate or severe FI (OR = 1.25; 95% CI: 1.03–1.53), with the association being even more significant for those with severe sarcopenia (OR = 1.88; 95% CI: 1.23–2.86). The results of the Brant test also showed that the proportional odds assumption was met for this model (*p* = 0.62).

## Discussion

Based on data from a nationally representative open cohort study of older Mexican adults, this study showed that moderate or severe FI is associated with a significant increase in longitudinal rates of sarcopenia and severe sarcopenia over a 12-year follow-up period. The incidence of severe FI was also associated with a significant increase in the incidence of sarcopenia and severe sarcopenia over a 4-year period. To the best of our knowledge, this is the first evidence of a prospective association between FI and sarcopenia.

The baseline prevalence of sarcopenia in our study was 12.9% and 2.7% in older adults with severe sarcopenia. These results are consistent with those of previous studies. One study conducted with adults aged 65 years and older in six LMICs reported a sarcopenia prevalence of 13.5% [5]. In addition, two recent systematic reviews have agreed with our findings. Petermann-Rocha et al. reported a prevalence of sarcopenia ranging from 10 to 27% [31], whereas Papapopulu et al. found a prevalence of sarcopenia of 11% in men and 9% in women [32]. For severe sarcopenia, a systematic review reported a weighted prevalence ranging from 2 to 9% [31].

In our study, the rates of sarcopenia and severe sarcopenia increased over the 12 years of follow-up. This trend differed according to the FI severity levels. Previous evidence has shown that muscle mass continuously decreases after reaching a peak (around 30 years), and the loss of the percentage of appendicular muscle mass is almost 5% for every 10 years of increase in age [8]. Our results are consistent with what Kim et al. reported on a constant and progressive decrease in the rates of longitudinal changes in muscle mass and muscle strength, which is accelerated from the age of 70–75 years [1]. Similarly, our results on the significant relationship between FI and sarcopenia reinforce the evidence about the role that modifiable behavioral factors, such as diet and lifestyle, play in the decline in muscle mass and strength [10].

Our findings support limited research on the association between FI and sarcopenia. One cross-sectional study with older adults from six LMICS found that severe FI was associated with a higher likelihood of sarcopenia (OR = 2.05, 95%CI = 1.12–3.73) [5]. Other studies have also reported a significant association between FI and probable sarcopenia, using poor grip strength as a proxy. Lee et al. reported that moderate and severe FI was significantly associated with poorer grip strength [16] and Lynch et al. observed that FI was associated with poorer grip strength (OR = 1.71; 95%CI = 1.08–2.71) [17]. However, a cross-sectional study conducted with older adults in Turkey found no significant association between FI and sarcopenia [33]. A potential explanation for this contrasting result is related to the study sample, as the Turkish study included people who attended primary care health centers. In contrast, the older adults in our study comprised a nationally representative sample and were interviewed in their homes.

One of the main mechanisms through which FI may be a determinant of health and disease is its impact on diet quality and sustainability [34]. As shown in previous studies, people with FI tend to switch to more affordable, energy-dense, processed food and less nutrient-dense foods, leading to poor diet quality and unsustainable food intake [35]. Leung et al. found that FI increased significantly over ten years (2007 and 2016) and this trend was associated with poorer diet quality, indicating that FI is associated with unhealthy dietary behaviors in older adults [36]. In the specific case of sarcopenia, FI is potentially associated with multiple micro- and macro-nutrient deficiencies, which in turn may increase the risk of sarcopenia [5].

A recent review of studies on nutritional interventions that analyzed the effects of micro and macronutrients and dietary patterns on muscle mass, muscle strength, and physical performance in older people highlighted the fundamental role of dietary protein intake [9]. Moreover, because older adults have a higher protein requirement per kg/body weight than young adults [37], adequate intake of good-quality protein is required to maintain physical performance and reduce the odds of sarcopenia [38]. Additionally, promising results have been identified for some specific nutrients, such as vitamin D, selenium, magnesium, and omega-3 fatty acids, although further studies are needed to determine the appropriate dose, frequency, and duration [9]. Finally, two recent systematic reviews (including one on LMICs) identified a significant association between diet quality and some of the components of sarcopenia, such as muscle mass, muscle strength, and physical performance [39, 40]. Both studies highlight the importance of adequate diet to maintain good physical performance in old age. However, they also highlighted the need for evidence generated from longitudinal studies. Given that FI is associated with low diet quality and low nutrient density, its association with sarcopenia can be explained through this pathway. However, further longitudinal studies are needed to explore the specific role of diet in sarcopenia onset.

The results of this study should be interpreted in light of the following limitations. First, although our results were generated using longitudinal data, this was an observational study, so it was not possible to draw causal conclusions. However, to the best of our knowledge, no previous studies have reported sarcopenia as a risk factor for FI. Nevertheless, it can be hypothesized that sarcopenic individuals may become more food insecure due to reduced mobility, economic constraints, or increased dependency on caregivers. The results of our sensitivity analysis supported this hypothesis. However, further longitudinal studies are needed to explore in greater depth the possible bidirectional relationship between sarcopenia and FI. Second, our measurement of FI was restricted to two single questions, leading to a potential misclassification in the assignment of FI levels, underestimating the severity of FI and its association with sarcopenia. Nevertheless, the results generated were in the expected direction, which is consistent with what has been previously reported. Additionally, we compared the classification obtained with our data with the most recent results on FI in the Mexican population. We used data from the National Health and Nutrition Survey of Mexico (ENSANUT for its acronym in Spanish), in which a validated questionnaire was applied to measure FI, Latin American and Caribbean Food Security Scale (ELCSA). The ENSANUT data showed the following proportions: 13.0% had moderate FI and 10.5% had severe FI [15]. Meanwhile, our data showed 7.8% with moderate insecurity and 10.7% with severe insecurity. That is, we classified individuals with severe FI correctly and underestimated those with moderate insecurity. Third, SAGE-Mexico does not contain data on diet, so the association between FI and diet quality is unknown, as well as the role of some dietary compounds that could account for sarcopenia (protein quality, vitamin D); therefore, residual confounding is present. Despite the latter, our study included fruit and vegetable consumption as potential confounders and as a proxy for diet quality.

Our findings have important implications for public health policies because FI has been identified as a critical public health problem in vulnerable populations, such as the older adult population [14]. For older adults, this situation is more critical given their socioeconomic situation since the need for food competes with other essential needs, such as medication, utilities, and transportation [41], making it difficult to meet their daily nutrient requirements. In particular, FI represents a double burden of malnutrition in older adults: undernutrition and overweight/obesity rates [42]. On the one hand, a restricted diet may reduce the quantity and quality of the diet, contributing to unintentional weight loss and undernutrition [42]. On the other hand, the same dietary restriction may imply excessive energy consumption but not critical nutrient-dense foods, contributing to obesity or overweight and metabolic dysfunction [34]. Moreover, because both conditions (undernutrition and overweight) have been identified as potential risk factors for sarcopenia, specific programs addressing food insecurity are needed to ensure access to nutritious foods. A multi-sectoral approach (government, nonprofit organizations, research institutions) is required to reach food security in all population groups [12], as well as to scale up strategies that have shown their effectiveness in high-income countries, such as food banks and food delivery -subsidized by the state- to older adults in their homes or increase the access of point of purchase nutritious foods [5]. Such strategies can be adapted and implemented in LMICs to tackle FI and reduce the incidence of sarcopenia in older adults.

In conclusion, the results of this study showed that moderate and severe FI are associated with increased rates of sarcopenia and severe sarcopenia over time. Addressing food insecurity should be a top public health priority in LMICs like Mexico. This challenge requires a collaborative effort among all sectors of society, including the government and nonprofit organizations, to ensure the effective implementation of tailored programs to alleviate the harmful consequences of FI.

## Electronic supplementary material

Below is the link to the electronic supplementary material.

Supplementary Material 1

## Data Availability

The raw dataset analyzed for this study is available at the following repository: https://www.who.int/data/data-collection-tools/study-on-global-ageing-and-adult-health/sage-waves.
