# Supplementary material for: Mid- and long-term associations between food insecurity and sarcopenia
Source: Aging Clin Exp Res. 2025 Mar 13;37(1):86. doi: 10.1007/s40520-025-02999-5 (PMC11903578; doi:10.1007/s40520-025-02999-5)
Supplement: Supplementary file 1 — Supplementary Material 1 [file 40520_2025_2999_MOESM1_ESM.docx]

**Supplementary material**

**Fig S1 Flow diagram for the analytical sample**

Baseline sample (Wave 1-2009)

n=2404

Excluded from follow-up (Wave 2)

273 dead

98 losses

Wave 2-2014

n=2651

Follow-up sample=2033

New sample=618

Excluded from follow-up (Wave 3)

242 dead

363 losses

Wave 3-2017

n=2046

Follow-up sample=1791

New sample=255

Excluded from follow-up (Wave 4)

184 dead

255 losses

Wave 4-2021

N=1961

Follow-up sample=1607

New sample=354

Excluded from follow-up sample missing values on some covariates: n=123

Analytical sample (all measurements)

n=1484

**Table S1. STROBE Statement—Checklist of items that should be included in reports of cohort studies**

|  | Item No | Recommendation | Page number |
| --- | --- | --- | --- |
| **Title and abstract** | 1 | (*a*) Indicate the study’s design with a commonly used term in the title or the abstract | 1 |
|  |  | (*b*) Provide in the abstract an informative and balanced summary of what was done and what was found | 2 |
| Introduction | | |  |
| Background/rationale | 2 | Explain the scientific background and rationale for the investigation being reported | 4 |
| Objectives | 3 | State specific objectives, including any prespecified hypotheses | 4-5 |
| Methods | | |  |
| Study design | 4 | Present key elements of study design early in the paper | 5 |
| Setting | 5 | Describe the setting, locations, and relevant dates, including periods of recruitment, exposure, follow-up, and data collection | 5 |
| Participants | 6 | (*a*) Give the eligibility criteria, and the sources and methods of selection of participants. Describe methods of follow-up | 5 |
|  |  | (*b*) For matched studies, give matching criteria and number of exposed and unexposed | N/A |
| Variables | 7 | Clearly define all outcomes, exposures, predictors, potential confounders, and effect modifiers. Give diagnostic criteria, if applicable | 5-6 |
| Data sources/ measurement | 8* | For each variable of interest, give sources of data and details of methods of assessment (measurement). Describe comparability of assessment methods if there is more than one group | 5-6 |
| Bias | 9 | Describe any efforts to address potential sources of bias | 7 |
| Study size | 10 | Explain how the study size was arrived at | 5 |
| Quantitative variables | 11 | Explain how quantitative variables were handled in the analyses. If applicable, describe which groupings were chosen and why | 7 |
| Statistical methods | 12 | (*a*) Describe all statistical methods, including those used to control for confounding | 7 |
|  |  | (*b*) Describe any methods used to examine subgroups and interactions | 9 |
|  |  | (*c*) Explain how missing data were addressed | N/A |
|  |  | (*d*) If applicable, explain how loss to follow-up was addressed | 5 |
|  |  | (*e*) Describe any sensitivity analyses | 7 |
| Results | | |  |
| Participants | 13* | (a) Report numbers of individuals at each stage of study—eg numbers potentially eligible, examined for eligibility, confirmed eligible, included in the study, completing follow-up, and analysed | Figure 1 |
|  |  | (b) Give reasons for non-participation at each stage | Figure 1 |
|  |  | (c) Consider use of a flow diagram | Figure 1 |
| Descriptive data | 14* | (a) Give characteristics of study participants (eg demographic, clinical, social) and information on exposures and potential confounders | 8 |
|  |  | (b) Indicate number of participants with missing data for each variable of interest | 8 |
|  |  | (c) Summarise follow-up time (eg, average and total amount) | 8-9 |
| Outcome data | 15* | Report numbers of outcome events or summary measures over time | 8-9 |
| Main results | 16 | (*a*) Give unadjusted estimates and, if applicable, confounder-adjusted estimates and their precision (eg, 95% confidence interval). Make clear which confounders were adjusted for and why they were included | 8-9 |
|  |  | (*b*) Report category boundaries when continuous variables were categorized | N/A |
|  |  | (*c*) If relevant, consider translating estimates of relative risk into absolute risk for a meaningful time period | N/A |
| Other analyses | 17 | Report other analyses done—eg analyses of subgroups and interactions, and sensitivity analyses | N/A |
| Discussion | | |  |
| Key results | 18 | Summarise key results with reference to study objectives | 9 |
| Limitations | 19 | Discuss limitations of the study, taking into account sources of potential bias or imprecision. Discuss both direction and magnitude of any potential bias | 11 |
| Interpretation | 20 | Give a cautious overall interpretation of results considering objectives, limitations, multiplicity of analyses, results from similar studies, and other relevant evidence | 9-10 |
| Generalisability | 21 | Discuss the generalisability (external validity) of the study results | 10-11 |
| Other information | | |  |
| Funding | 22 | Give the source of funding and the role of the funders for the present study and, if applicable, for the original study on which the present article is based | 3 |

**Table S2. Longitudinal associations of sarcopenia and food insecurity**

| **Outcome: food insecurity** | OR | CI 95% | | p-value |
| --- | --- | --- | --- | --- |
| Sarcopenia (reference category: no) |  |  |  |  |
| Sarcopenia | 1.25 | 1.03 | 1,53 | 0.02 |
| Severe sarcopenia | 1.88 | 1.23 | 2.86 | <0.01 |
| Brant test* (p=0.62) |  |  |  |  |

* Brant test for proportional odds assumption
